# The Effect of Biliary Decompression on Bacterial Translocation in Jaundiced Rats

**DOI:** 10.1155/1993/69283

**Published:** 1993

**Authors:** Jin Wen Ding, Roland Andersson, Vasile Soltesz, Roger Willén, Steffen Loft, Henrik E. Poulsen, Håkan Pärsson, Kjell Olsson, Stig Bengmark

**Affiliations:** 1 Department of Surgery Lund University Lund Sweden; 2 Department of Microbiology Lund University Lund Sweden; 3 Department of Pathology Lund University Lund Sweden; 4 Department of Pharmacology Copenhagen University Copenhagen Denmark; 5 Department of Zoology Lund University Lund Sweden; 6 Department of Surgery Lund University Lund S-221 85 Sweden

## Abstract

Patients with obstructive jaundice are prone to septic complications after biliary tract operations. Restoring bile flow to the intestine may help to decrease the complication rate. The present study is aimed at evaluating the effect of biliary decompression on bacterial translocation in jaundiced rats.

Sixty-six male Sprague-Dawley rats were randomly allocated to six groups subjected to common bile duct ligation (CBDL) and transection (groups 2–6) or sham operation (group 1). In groups and 2 the incidence of enteric bacterial translocation was determined 2 weeks after sham operation or CBDL. In groups 3–6, biliary decompression was achieved by performing a choledochoduodenostomy after 2 weeks of biliary decompression. Bacterial translocation was then studied 1,2,3 and 5 weeks following biliary decompression.

The rate of bacterial translocation to mesenteric lymph nodes in obstructive jaundice was significantly higher as compared with controls, and decreased with time to nil three weeks following biliary decompression. The incidence of bacterial translocation was closely correlated (*r* = 0.844; *p* = 0.034) with serum alkaline phosphatase activity and seemed to fit with the morphological changes noted in the small intestine. The decrease in bacterial translocation, however, lags behind the recovery of liver function as measured by routine liver function tests and antipyrine clearance.

Obstructive jaundice thus promotes bacterial translocation in the rat. Biliary decompression gradually decreases the rate of bacterial translocation.


HPB Surgery, 1993, Vol. 7, pp. 99-110
Reprints available directly from the publisher
Photocopying permitted by license only
© 1993 Harwood Academic Publishers GmbH
Printed in the United States of America

### THE EFFECT OF BILIARY DECOMPRESSION ON BACTERIAL TRANSLOCATION IN JAUNDICED RATS

JIN WEN DING, ROLAND ANDERSSON, VASILE SOLTESZ[1], ROGER WILLÉN[2], STEFFEN LOFT[3], HENRIK E. POULSEN[3], HÅKAN PÄRSSON, KJELL OLSSON[4], and STIG BENGMARK

*Departments of Surgery, Microbiology[1], Pathology[2] and Structural Zoology[4], Lund University, Lund, Sweden, and Department of Pharmacology[3], Copenhagen University, Copenhagen, Denmark.*

*(Received 27 November 1992)*

Patients with obstructive jaundice are prone to septic complications after biliary tract operations. Restoring bile flow to the intestine may help to decrease the complication rate. The present study is aimed at evaluating the effect of biliary decompression on bacterial translocation in jaundiced rats.

Sixty-six male Sprague-Dawley rats were randomly allocated to six groups subjected to common bile duct ligation (CBDL) and transection (groups 2–6) or sham operation (group 1). In groups 1 and 2 the incidence of enteric bacterial translocation was determined 2 weeks after sham operation or CBDL. In groups 3–6, biliary decompression was achieved by performing a choledochoduodenostomy after 2 weeks of biliary decompression. Bacterial translocation was then studied 1,2,3 and 5 weeks following biliary decompression.

The rate of bacterial translocation to mesenteric lymph nodes in obstructive jaundice was significantly higher as compared with controls and decreased with time to nil three weeks following biliary decompression. The incidence of bacterial translocation was closely correlated ($r = 0.844$; $p = 0.034$) with serum alkaline phosphatase activity and seemed to fit with the morphological changes noted in the small intestine. The decrease in bacterial translocation, however, lags behind the recovery of liver function as measured by routine liver function tests and antipyrine clearance.

Obstructive jaundice thus promotes bacterial translocation in the rat. Biliary decompression gradually decreases the rate of bacterial translocation.

KEY WORDS: Obstructive jaundice, cholestasis, septicemia, bile, choledochoduodenostomy

### INTRODUCTION

Obstructive jaundice is frequently associated with septic complications following biliary tract surgery[1,2]. The mechanisms for this clinical phenomenon are not entirely clear. Frequently, enteric, gram negative bacteria have been isolated from the infectious focus (mainly wound, bile or abscess)[3,4]. Recently, Deitch, *et al.* showed that bacterial translocation (i.e. enteric bacteria crossing the intestinal wall and invading extra-intestinal sites) occurred in obstructive jaundice in mice[5]. Bailey[6] and Koscár *et al.*[7] have reported that jaundiced subjects are prone to endotoxaemia, due to the increased absorption and the decreased hepatic clearance

Address correspondence to: Roland Andersson, M.D., Ph.D. Department of Surgery, Lund University, S-221 85 Lund, Sweden.

capacity of the reticuloendothelial system (RES) in obstructive jaundice[8-10]. The recovery of intestinal bile flow has been considered to be crucial in the prevention of endotoxaemia and relief of the biliary obstruction has also been demonstrated to be important in the recovery of RES function[11]. Internal biliary drainage could both conduct bile flow back to the intestine and render relief of jaundice in biliary obstruction.

The purpose of the present study is to evaluate the effect of internal biliary drainage, by choledochoduodenostomy, on the incidence of bacterial translocation in rats with obstructive jaundice.

### MATERIALS AND METHODS

### *Animals and Experimental Design*

Sixty-six male Sprague-Dawley (Møllegaard Ltd., Skensved, Denmark) rats, weighing 270–350g, were randomly allocated to six groups, in which groups 2–6 were subjected to obstructive jaundice by ligating the common bile duct, whereas group 1 underwent sham operation. The animals were kept in controlled temperature (22 °C), humidity and 12-hour light/dark cycles and allowed rat pellets (ALTROMIN NR.1324, Altromin Spezialfutterwerke GHMB, Germany) and tap water ad libitum before and after the operations. The bacterial translocation study was performed 2 weeks following common bile duct ligation (CBDL, group 2) or sham operation (group 1) as well as 1, 2, 3 and 5 weeks after biliary decompression (BD) (groups 3–6).

### *Surgical Procedures*

An upper-midline abdominal incision was made through which the common bile duct was identified, double ligated with 5-0 silk (ETHICON, GmbH, Germany) and transected between the ligatures[12]. Sham operation was performed by mobilizing the common bile duct from the surrounding tissues without ligation and division. Internal biliary drainage was then achieved by performing a choledochoduodenostomy. A longitudinal incision (0.8 cm) was made in the duodenum and the dilated common bile duct remnant, respectively. The side to side anastomosis was established by a continuous single suture layer with 6–0 silk (ETHICON, GmbH, Germany). All operations were carried out under sterile conditions using light ether anaesthesia.

### *Liver Function Tests*

Blood samples for the determination of serum concentrations of bilirubin, alanine aminotransferase (ALAT), aspartate aminotransferase (ASAT) and alkaline phosphatase (ALP), were taken at the time of bacterial translocation studies. Bilirubin was determined in accordance with the method of Kuffer *et al.*[13] and the other enzymes were determined using the methods of the Committee on Enzymes of the Scandinavian Society of Clinical Chemistry and Clinical Physiology[14,15].

*Plasma Antipyrine Clearance*

Plasma antipyrine clearance, a determinant of microsomal function of hepatocytes, was analysed by a single sample method assay[16]. Four mg of antipyrine (Sigma Chemical Co., St Louis, Mo., USA) were dissolved in sterile saline and injected intravenously (penile vein) 5 hours before performing common bile duct ligation, biliary decompression or bacterial translocation studies 2, 3 and 5 weeks after biliary decompression. Five hours after the injection, blood samples for determining antipyrine clearance were taken into heparinized tubes and centrifuged at 2000 rpm for 15 min, after which plasma was removed and frozen at -20 °C. Antipyrine was analysed by high performance liquid chromatography (HPLC) as described previously[16]. The antipyrine plasma clearance was calculated by using the one-sample method as

$$\text{Plasma antipyrine clearance} = 0.66\text{b.w.x}[\ln(\text{D}/0.66\text{b.w.})-\ln(c_t)]/t$$

where D is the antipyrine dose, 0.66b.w. is the antipyrine apparent volume of distribution[16] and $c_t$ is the concentration at the sampling time $t$[16].

*Bacteriological Study*

A modification of the methodology described by Deitch *et al.*[5] and Jones II *et al.*[17] was used in the present study. The abdomen was shaved, twice soaked with chlorhexidine in spirit (KabiVitrum AB, Stockholm, Sweden) and aseptically opened with sterile instruments. Aliquots of 0.5 ml blood from the portal vein and inferior vena cava were transferred into tubes containing 2 ml of Brain Heart Infusion (BHI) broth (Difco Laboratories, Detroit, Michigan, USA). The liver, spleen and mesenteric lymph nodes were removed and placed in preweighed tubes containing Tryptic Soy Broth (TSB) (Oxoid, Basingstoke, Hampshire, UK). The peritoneal cavity and the peritoneal viscera were swabbed with sterile cotton tipped applicator sticks and cultured in BHI broth in order to demonstrate accidental bacterial contamination. Finally, the caecum was opened and 0.5 ml caecal content was inoculated into TSB.

Aliquots of 0.2 ml of homogenized organ samples and the supernatant of the caecal content after settlement of vortexing were inoculated into 3 ml BHI broth, and plated on blood agar and McConkey agar plates (Oxoid, Basingstoke) for the culture of aerobic and microaerophilic bacteria. Bacto-Rogosa SL broth (Difco Laboratories) was used for culture of *Lactobacilli* and anaerobic bacteria. Aerobic and anaerobic cultures of blood were incubated at 37 °C for 7 days and read daily. If any macroscopic bacterial growth was noted, subcultures on blood agar were performed. All organ samples were incubated aerobically at 37 °C overnight, as well as anaerobically at 37 °C for 7 days before being discarded. Enteric Gram-negative bacteria were identified by using the API 20 system (BioMeriux SA, Marcy - 1′ Etoile, France), while all other aerobic, microaerophilic and anaerobic bacteria were identified with standard procedures[18]. For identification of *Lactobacillus acidophilus*, API 50CH (Analytab Products Inc., Plainview, New York, USA) was used.

### *Morphological Study*

The terminal ileum was removed after the animals were killed. The specimens were fixed in 10% formalin in 0.15 M phosphate buffer, pH7.2 (effective osmolar pressure 300 m OSM), embedded in paraffin, stained with haematoxylin and eosin and examined under the light microscope.

### *Scanning Electron Microscopy (SEM)*

The terminal ileum was removed from both controls and the jaundiced animals immediately after the animals were killed. The small intestine was cannulated and carefully rinsed with lactated Ringer's solution. The specimens were then drip-fixed in 2.5% Glutaraldehyde in phosphate buffer. Further fixation was achieved by immersion in 2.5% Glutaraldehyde for 24 hours and subsequently the specimens were extensively rinsed in Millonig's phosphate solution. The specimens were prepared for scanning electron microscopy (SEM) by dehydration in ethanol and transferred to the critical point drier. The mounted specimens were sputtered in a polaron 5400 sputtercoater unit with gold-palladium and studied in a JEOL T 330 SEM (Tokyo, Japan).

### *Statistical Methods*

One way analysis of variance (ANOVA) was used for parametric data, while the comparison of bacterial translocation rates between the groups was calculated by Chi-square with Yates' correction. All values were expressed as mean ± SEM. A probability less than 5% was considered statistically significant.

### RESULTS

All rats with common bile duct ligation were jaundiced and bile was present in the urine. No bilio-intestinal patency could be found in jaundiced rats when performing choledochoduodenstomy or following the bacterial translocation study (sacrifice).

### *Liver Function*

Serum levels of bilirubin, alanine aminotransferase, aspartate aminotransferase and alkaline phosphatase were significantly elevated ($p < 0.001$) in animals with 2 weeks of biliary obstruction as compared with controls and biliary decompressed animals, and decreased to normal levels one week following biliary decompression, without significant difference ($p > 0.05$) among the decompressed animals (Table 1). The antipyrine clearance rate decreased 20% ($p < 0.01$) after 2 weeks of biliary obstruction and normalized ($p < 0.01$) within 2 weeks following biliary decompression, after which the clearance rate did not further increase (Table 2).

### *Bacterial Translocation*

Mesenteric lymph nodes (MLNs) were the sole source of bacterial growth. The incidence of positive bacterial cultures in MLNs in animals with 2 weeks of

**Table 1** Liver function tests (mean±SEM)

| *Groups* | *Bilirubin (μ mol/l)* | *ALAT (μ Kat/l)* | *ASAT (μ Kat/l)* | *ALP (μ Kat/l)* |
|---|---|---|---|---|
| Controls | 2.50±0.61 | 1.00±0.11 | 1.23±0.06 | 6.76±1.08 |
| Jaundice | 116.71±10.03‡ | 2.04±0.29‡ | 6.87±0.99‡ | 18.64±1.22‡ |
| 1W BD | 3.60±0.41 | 0.74±0.06 | 1.31±0.07 | 10.60±1.04 |
| 2W BD | 3.31±1.04 | 0.83±0.05 | 1.50±0.26 | 8.67±1.07 |
| 3W BD | 3.51±0.38 | 0.94±0.07 | 1.56±0.10 | 9.09±0.67 |
| 5W BD | 2.85±0.73 | 1.03±0.08 | 1.65±0.18 | 7.80±0.34 |

‡ = p<0.001 as compared with controls and all biliary decompressed groups.
1W BD: one week following biliary decompression. 2W BD: Two weeks following biliary decompression.
3W BD: Three weeks following biliary decompression. 5W BD: Five weeks following biliary decompression.

**Table 2** Antipyrine clearance (mean±SEM)

| *Groups* | *Antipyrine Clearance (ml/min/kg)* |
|---|---|
| Normal | 7.11±0.32 |
| Jaundice | 5.68±0.17† |
| 2W BD | 7.28±0.43 |
| 3W BD | 7.04±0.62 |
| 5W BD | 6.81±0.42 |

† = p<0.01 as compared with controls and biliary decompressed groups.
2W BD: Two weeks following biliary decompression. 3W BD: Three weeks following biliary decompression.
5W BD: Five weeks following biliary decompression.

obstructive jaundice (47%) was significantly higher ($p < 0.05$) than in controls (10%). The rate of positive cultures in MLNs consistently decreased from (29%) 1 week after biliary decompression to nil both 3 and 5 weeks following the relief of obstructive jaundice (Table 3). The bacteria most frequently identified from the MLNs included *Escherichia coli, Enterobacter sp., Pseudomonas sp., Proteus sp.* and in some instances *Staphylococcus sp.* Thus, enteric bacteria dominated culture findings in MLNs. One positive culture from the liver was noted in all groups except for group 5 (3W BD). Two positive bacterial cultures from the spleen and one positive blood culture were found in group 3 (1W BD).

Caecal levels of *E. coli* did not significantly differ among the various groups ($p >$ 0.05), though the level in animals with 2 weeks of biliary obstruction tended to be highest. Viable counts of aerobes and microaerobes in rats 1 week after biliary decompression were significantly higher ($p < 0.01$) as compared with both controls, jaundiced and all other biliary decompressed animals. The counts of *Lactobacilli* in jaundiced and decompressed animals were lower than in controls, though without statistical significance ($p > 0.05$). A tendency of recovery of *Lactobacilli* levels after biliary decompression was noted (Table 4).

The rate of bacterial translocation, defined as positive MLNs cultures, was evaluated using Pearson's correlation in order to investigate if any correlation with changes in liver function in obstructive jaundice existed. It was found that the

**Table 3** Positive bacterial cultures in various organs and blood

| *Groups* | | *MLNs* | *Liver* | *Spleen* | *Blood* |
|---|---|---|---|---|---|
| Controls | (n = 10) | 1/10 | 1/10 | 0/10 | 0/10 |
| Jaundice | (n = 15) | 7/15* | 1/15 | 0/15 | 0/15 |
| 1W BD | (n = 14) | 4/14 | 1/14 | 2/14 | 1/14 |
| 2W BD | (n = 9) | 2/9 | 1/9 | 0/9 | 0/9 |
| 3W BD | (n = 9) | 0/9 | 0/9 | 0/9 | 0/9 |
| 5W BD | (n = 9) | 0/9 | 1/9 | 0/9 | 0/9 |

* = p>0.05 when compared with controls. 1W BD: 1 week of biliary decompression. 2W BD: 2 weeks of biliary decompression. 3W BD: 3 weeks of biliary decompression. 5W BD: 5 weeks of biliary decompression. MLNs: Mesenteric lymph nodes.

**Table 4** Caecal population levels of bacteria (CFU/g) (mean±SEM)

| *Groups* | | *Escherichia coli* $\times 10^4$ | *Aerobes/Microaerobes* $\times 10^2$ | *Lactobacilli* $\times 10^2$ |
|---|---|---|---|---|
| Controls | (n = 10) | 14.8±5.4 | 13.3±6.0 | 43.6±26.1 |
| Jaundice | (n = 15) | 748.0±541.0 | 4.3±0.5 | 3.2±0.9 |
| 1W BD | (n = 14) | 23.5±2.5 | 25.5±1.4† | 2.0±0.5 |
| 2W BD | (n = 9) | 15.5±3.7 | 12.7±1.3 | 3.6±0.8 |
| 3W BD | (n = 9) | 11.0±2.4 | 14.2±1.3 | 20.8±3.1 |
| 5W BD | (n = 9) | 19.9±2.3 | 13.5±0.8 | 22.7±4.5 |

† = p>0.01 as compared with all other groups.
1W BD: one week following biliary decompression. 2W BD: Two weeks following biliary decompression. 3W BD: Three weeks following biliary decompression. 5W BD: Five weeks following biliary decompression.

bacterial translocation rate significantly correlated ($R$ = 0.844; $p$ = 0.034) with alkaline phosphatase activity and that it tended to correlate, though not significantly, with bilirubin, alanine aminotransferase and aspartate aminotransferase (Table 5).

### *Intestinal Morphology*

The intestinal specimens from animals with 1, 3 and 5 weeks of biliary decompression were examined with light microscopy. Dilated lymph vessels and oedematous subepithelial regions with infiltration of inflammatory cells in the villi were demonstrated in jaundiced animals, findings that were reduced with time after biliary decompression (Figure 1).

### *Scanning Electron Microscopy*

Numerous *Lactobacilli* adherent to the surfaces of the intestinal epithelial cells could be demonstrated using SEM in control animals, while only sparse *Lactobacilli* was noted on the surfaces of the epithelial cells in jaundiced rats (Figure 2).

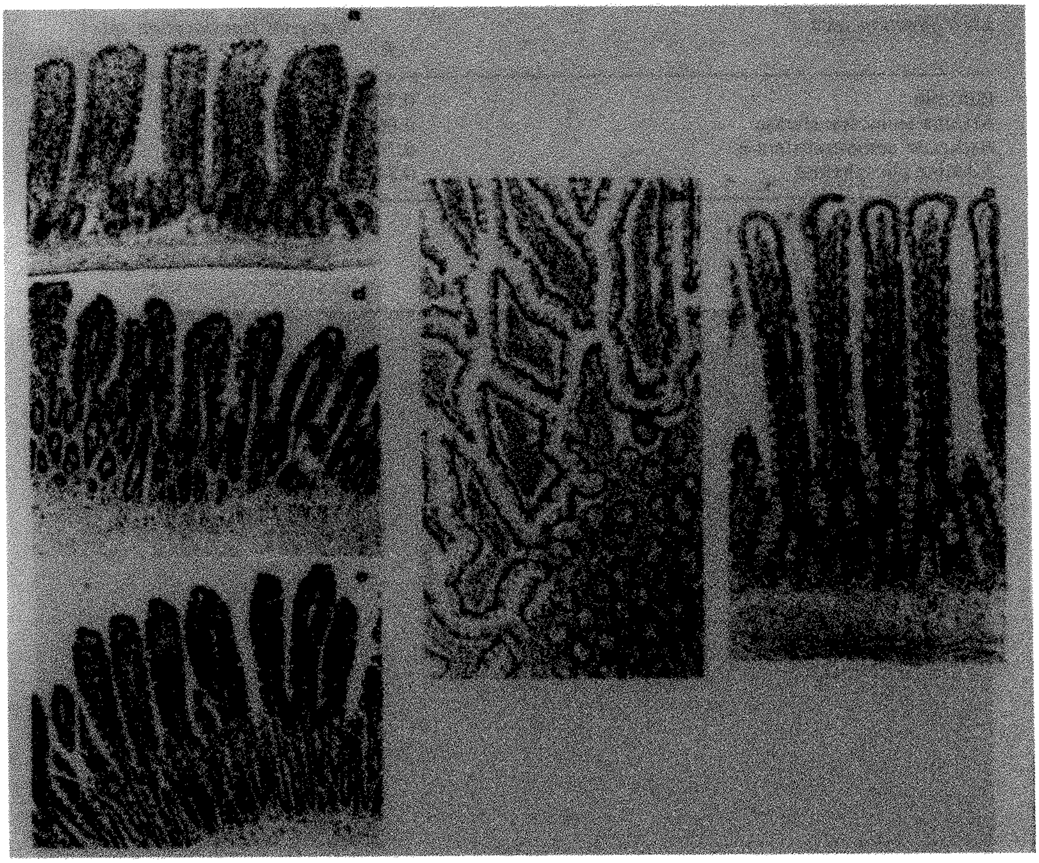

**Figure 1a** Normal small intestine with high slender villi and no oedema (H&E × 25).

**Figure 1b** Small intestine from jaundiced animals (2 weeks). The subepithelial edema of the villi is obvious with dilated lymph vessels (H&E × 75).

**Figure 1c** The oedematous subepithelial region on the tip of the villi with enhancement of inflammatory cell infiltration one week after biliary decompression (H&E × 25).

**Figure 1d** Small intestinal villi with dilated lymph vessels and infiltration of inflammatory cells in the stroma three weeks following relief of obstructive jaundice (H&E × 25).

**Figure 1e** Reduction of the dilatation lymph vessels and infiltration of inflammatory cells in the stroma is noted five weeks following biliary decompression.

**Table 5** Correlation between the incidence of bacterial translocation and liver function

| *Liver function tests* | *Bacterial translocation rate* *R* | *p values* |
|---|---|---|
| Bilirubin | 0.775 | 0.070 |
| Alanine aminotransferase | 0.062 | 0.186 |
| Aspartate aminotransferase | 0.745 | 0.089 |
| Alkaline phosphatase | 0.844 | 0.034* |

R: Correlation coefficient. * = p>0.05.

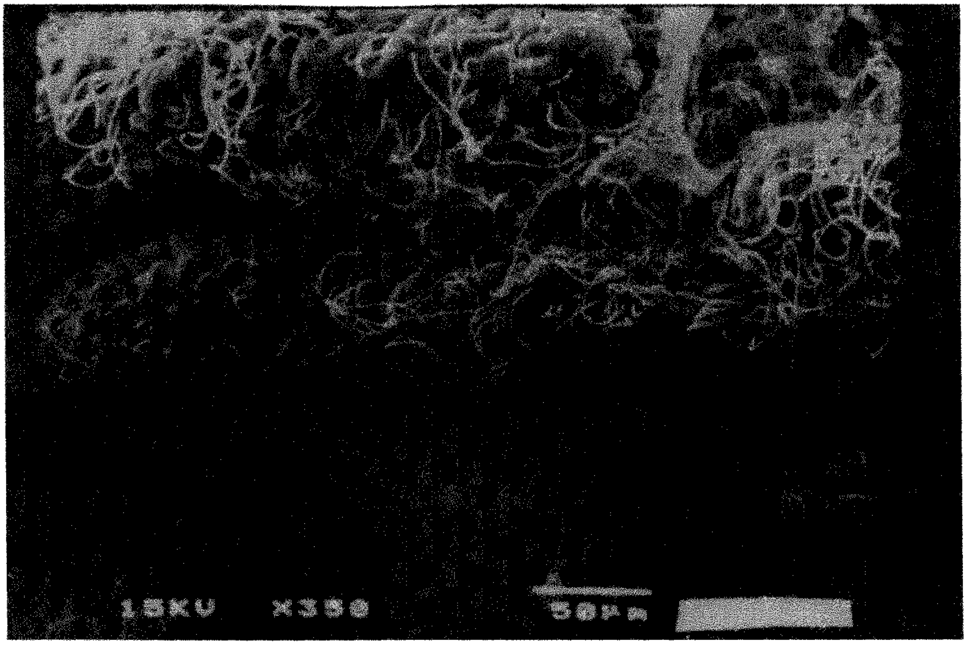

**Figure 2a** Numerous *Lactobacilli* adherent to the surfaces of the intestinal epithelial cells in control animals (SEM × 2000).

### DISCUSSION

Biliary tract surgery in jaundiced patients is frequently associated with septic complications[1–3], such as biliary sepsis, wound infection, intraabdominal abscess formation, and renal insufficiency[19,20]. Gunn[3] reported that wound infection, subphrenic abscess and septicemia are 4 times more common in patients with infected bile than in patients in whom the bile is sterile. Bacteriaemia and septicemia in obstructive jaundice were usually demonstrated to be secondary to both the occurrence of infected bile and increased biliary pressure[21]. The precise aetiology of infectious complications in jaundiced patients without biliary infection is, however, still uncertain.

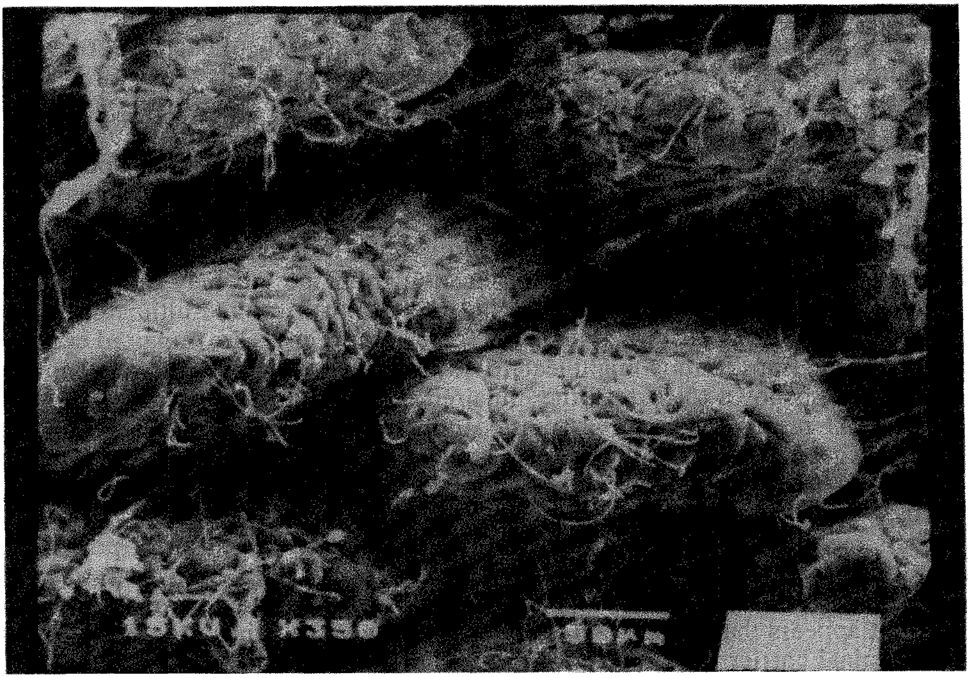

**Figure 2b** Sparse *Lactobacilli* attached to the surfaces of the intestinal epithelail cells in jaundiced animals (SEM × 2000).

The exact causes of bacterial translocation in obstructive jaundice could not be determined by the current study. Factors promoting bacterial translocation from the gut include disruption of the ecology of the indigenous intestinal flora[22], compromised host immunity[23] and physical disruption of the gut mucosal barrier[24]. The high incidence of bacterial translocation in jaundiced animals might relate to the disrupted ecology of the intestinal flora, which might at least partly, be due to the absence of intestinal bile flow. Furthermore, viable counts of *Lactobacilli* in jaundiced animals decreased, though not statistically significantly. Enteric anaerobic bacteria outnumber aerobic and facultative bacteria by 100:1 or 1000:1 and might function to control colonization and translocation of facultative bacteria by occupying sites of adhesion for facultative bacteria[25,26] and releasing an antimicrobial substance[27]. In the present study a marked decrease in the number of *Lactobacilli* adherent to the intestinal surface during biliary obstruction was demonstrated by scanning electron microscopy. Reestablishment of the biliodigestive continuity both restores intestinal bile flow and improves liver function. Furthermore, bile acids have been demonstrated to inhibit bacterial growth both *in vivo* and *in vitro*[28,29], in the current study the incidence of bacterial translocation gradually, but slowly decreased following biliary drainage by performing a choledochoduodenostomy. The incidence of bacterial translocation after relieving obstructive jaundice for one week was still high (29%). Long term obstructive jaundice and subsequent operative trauma might act as a major insult leading to suppression of host immunity and resultant bacterial overgrowth. No bacterial translocation was noted three weeks after relief of biliary obstruction. The tendency towards recovery of intestinal *Lactobacilli* counts and diminished rate of bacterial translocation after biliary decompression implies the importance of biliary decompression in order to reduce the susceptibility to septic complications.

Compromised host immunity promotes bacterial translocation in obstructive jaundice. Nonspecific[30] and specific cellular immunity[31], as well as reticuloendothelial dysfunction[8-10] have all been reported in experimental biliary obstruction. The clinical significance of bacterial translocation and transient bacteriaemia, however, depends upon several factors like the integrity of the host defense and invasiveness of the microorganisms. Bacterial translocation from the gut may thus be of no clinical significance if host defenses are adequate to control and ultimately eradicate the translocating bacteria. However, if the host defenses are impaired enough, then bacterial translocation might lead to a systemic infection[31]. Biliary decompression has been shown to reverse the impaired immunity in biliary obstruction[31,33]. The present results on bacterial translocation supports previous findings by this group[11], where RES function was demonstrated to be in a depressed state still one and two weeks after biliary decompression, recovering in the third week after relief of obstructive jaundice.

The integrity of the intestinal mucosal barrier function is critical for preventing microorganisms from invading. Subepithelial oedema, dilated lymph vessels and inflammatory cell infiltration were noted in obstructive jaundice in the present study. It appears that these pathological changes might diminish the efficiency of the intestinal barrier function, though intestinal integrity otherwise is maintained. The histological changes found in the intestine following biliary obstruction and initially following biliary decompression, might thus contribute to an impaired local host defence and increased permeability. These pathological alterations reduced with time after biliary decompression. Further studies are warranted to investigate the intestinal morphological changes and their implications in obstructive jaundice.

Liver function, especially the activity of alkaline phosphatase, correlated with the incidence of bacterial translocation. Since the liver is involved in a variety of metabolic processes, either liver disease[34,35] or obstructive jaundice[36], could alter various metabolic mechanisms in the body, which might then affect intestinal function and lead to bacterial translocation. Antipyrine clearance capacity is a measurement of liver microsomal function and correlates with other "vital hepatic functions" of non-microsomal nature such as galactose elimination capacity and the capacity for urea-N-synthesis[37]. Auranen *et al.*[38] reported a significant decrease in glucose-6-phosphatase activity, in aminopyrine demethylation, hexobarbital metabolism and cytochrome P-450 content, indicating impairment of liver microsomal function in obstructive jaundice. Antipyrine clearance capacity returned to normal levels two weeks after biliary decompression in our study. It seems that the recovery of liver function after biliary decompression preceded the reduction of the rate of bacterial translocation.

In conclusion, bacterial translocation occurs in obstructive jaundice in the rat and decreases after biliary decompression.

*Acknowledgement*

This study has been supported by grants from the Medical Faculty, Lund University and the Lars Hierta Memorial Foundation.

### *References*

1. Blamey, S.L., Fearson, K.C.H., Gilmour, W.H., Osborne, D.H. and Carter, D.C. (1983) Prediction of risk in biliary surgery. *The British Journal of Surgery*, **70**, 590–595
2. McPherson, G.A.D., Benjamin, I.S., Hodgson, H.J.F., Bowley, N.B., Allison, D.J. and Blumgart, L.H. (1982) Percutaneous transhepatic drainage in obstructive jaundice: advantage and problems. *The British Journal of Surgery*, **69**, 261–264
3. Gunn, A.A. (1982) Antimicrobial prophylaxis in biliary surgery. *World Journal of Surgery*, **6**, 301–305
4. Wells, G.R., Taylor, E.W., Lindsay, G., Morton, L. and the West of Scotland Surgical Infection Study Group (1989) Relationship between bile colonization, high-risk factors and postoperative sepsis in patients undergoing biliary tract operations while receiving a prophylactic antibiotics. *The British Journal Surgery*, **76**, 374–377
5. Deitch, E.A., Sittig, K., Li, M., Berg, R. and Specian, R.D. (1990) Obstructive jaundice promotes bacterial translocation from the gut. *American Journal of Surgery*, **159**, 79–84
6. Bailey, M.E. (1976) Endotoxin, bile salts and renal function in obstructive jaundice. *The British Journal Surgery*, **63**, 774–778
7. Kocsár, L.T., Bertók, L., Vartéresz, V. (1969) Effect of bile acids on the intestinal absorption of endotoxin in rats. *Journal of Bacteriology*, **100**, 220–223
8. Ding, J.W., Andersson, R., Norgren, L., Stenram, U. and Bengmark, S. (1992) The influence of biliary obstruction and sepsis on reticuloendothelial function in rats. *The European Journal of Surgery*, **158**, 157–164
9. Pain, J.A. (1987) Reticuloendothelial function in obstructive jaundice. *The British Journal Surgery*, **74**, 1091–1094
10. Drivas, G., James, O. and Wardle, N. (1976) Study of reticuloendothelial phagocytic capacity in patients with cholestasis. *British Medical Journal*, **1**, 1568–1569
11. Ding, J.W., Andersson, R., Stenram, U., Lundquist, A. and Bengmark, S. (1992) The effect of biliary decompression on reticuloendothelial function in jaundiced rats. *The British Journal Surgery*, **79**, 648–652
12. Trams, E.G. and Symeonidis, A. (1957) Morphological and functional changes in the liver of rats after ligation or excision of the common bile duct. *American Journal Pathology*, **33**, 13–27
13. Kuffer, H., Rictherich, R., Pehein, E. and Colombo, J.P. (1974) Die Bestimmung des Bilirubin in Plasma und Serum als Azobilirun mit dem Greiner Electronic Selective Analyser GSA II. *Zeitschrift fur Klinische Chemie und Klinische Biochemie*, **12**, 294–302
14. Scandinavian Committee on Enzymes: (1974) Recommended methods for the determination of four elements in the blood. *Scandinavian Journal of Clinical & Laboratory Investigation*, **33**, 291–306
15. Scandinavian Committee on Enzymes (1981) Experience with Scandinavian recommended method for determination of enzymes in blood. *Scandinavian Journal of Clinical & Laboratory Investigation*, **41**, 107–116
16. Pilsgaar, H. and Poulsen, H.E. (1984) A one-sample method for antipyrine clearance determination in rats. *Pharmacology*, **29**, 110–116
17. Jones II, W.G., Minei, J.P., Barber, A.E., Rayburn, J.L., Fahey III, T.J., Shires III, G.T. and Shires, G.T. (1990) Bacterial translocation and intestinal atrophy after thermal injury and burn wound sepsis. *Annals of Surgery*, **211**, 399–405
18. *Manual of clinical microbiology*, edited by A. Balows (1991) American Society of Microbiology, Washington, D.C.
19. Allison, M.E.M., Prentice, C.R.M., Kennedy, A.C. and Blumgart, L.H. (1979) Renal function and other factors in obstructive jaundice. *The British Journal Surgery*, **66**, 392–397
20. Thompson, J.N., Edwards, W.H., Winearls, C.G., Blenkharn, J.I., Benjamin, I.S. and Blumgart, L.H. (1987) Rental impairment following biliary tract surgery. *The British Journal Surgery*, **74**, 843–847
21. Carlson, H.C. (1970) Percutaneous transhepatic cholangiography. *Medical Clinics of North America*, **54**, 875–879
22. Berg, R.D. and Garlington, A.W. (1979) Translocation of certain indigenous bacteria from the gastrointestinal tract to the mesenteric lymph nodes and other organs in a gnotobiotics mouse model. *Infection and Immunity*, **23**, 403–411
23. Berg, R.D. (1983) Bacterial translocation from the gastrointestinal tracts of mice receiving immunosuppressive chemotherapeutic agents. *Current Microbiology*, **8**, 285–292
24. Morehouse, J., Specian, R., Stewart, J., Berg, R.D. (1986) Promotion of the translocation of indigenous bacteria of mice from the GI tract by oral ricinoleic acid. *Gastroenterology*, **91**, 673–682

25. Moore, W.E.C. and Holdeman, L.V. (1975) Discussion of current bacteriological investigations between intestinal flora, diet and colon cancer. *Cancer Research*, **35**, 3418–3412
26. Van der Waaij, D., Berghuis-De Vries, J.M. and Lekkerkerk-Van der Wees (1971) Colonization resistence of the digestive tract in conventional and antibiotic-treated mice. *Journal of Hygiene*, **69**, 405–411
27. Silva, M., Jacobus, N.V., Deneke, C. and Gorbach, S.L. (1987) Antimicrobial substance from a human *Lactobacillus* strain. *Antimicrobial Agents and Chemotherapy*, **31**, 1231–1233
28. Floch, M.H., Gershengoren, W., Elliott, S. and Spiro, H.M. (1971) Bile acid inhibition of the intestinal microflora. A function for simple bile acids? *Gastroenterology*, **61**, 228–233
29. Williams, R.C., Showalter, R. and Kern, F. (1975) *In vivo* effect of bile salts and cholestyramine on intestinal anaerobic bacteria. *Gastroenterology*, **69**, 483–491
30. Roughneen, P.T., Drath, D.B., Kulkarni, A.D. and Rowlands, B.J. (1987) Impaired nonspecific cellular immunity in experimental cholestasis. *Annals of Surgery*, **206**, 578–582
31. Roughneen, P.T., Gouma, D.J., Kulkarni, A.D., Fanslow, W.F. and Rowlands, B.J. (1986) Impaired specific cell-mediated immunity in experimental biliary obstruction and its reversibility by internal biliary drainage. *Journal of Surgical Research*, **41**, 113–125
32. Deitch, E.A. (1990) Bacterial translocation: Is it of clinical significance? *Gastroenterology*, **98**, 243–244
33. Thompson, R.L.E., Hoper, M., Diamond, T. and Rowlands, B.J. (1990) Development and reversibility of T lymphocyte dysfunction in experimental obstructive jaundice. *The British Journal Surgery*, **77**, 1229–1232
34. Record, C.O., Alberti, K.G.M.M. and Williamson, D.H. (1972) Metabolic studies in experimental liver disease resulting from D(+)-galactosamine administration. *Biochemical Journal*, **130**, 37–44
35. Mortiaux, A. and Dawson, A.M. (1961) Plasma free fatty acid in liver disease. *Gut*, **2**, 304–309
36. Younes, R.N., Vydelingum, N.A., DeRooij, P., Scognamiglio, F., Andrade, L., Posner, M.C. and Brennan, M.F. (1991) Metabolic alterations in obstructive jaundice: Effect of duration of jaundice and bile-duct decompression. *HPB Surgery*, **5**, 35–48
37. Hansen, B.A. and Poulsen, H.E. (1986) The capacity of urea-N-synthesis as a quantitative measure of the liver mass in rats. *Journal of Hepatology*, **2**, 468–475
38. Auranen, A., Ahonen, J. and Hvitfelt, J. (1973) Effect of biliary obstruction on liver microsomes in the rat. *European Surgical Research*, **5**, 228–232

*(Accepted by L. Blumgart, 7 October 1992)*

